# eHealth Communication With Clients at Community-Based HIV/AIDS Service Organizations in the Southern United States: Cross-Sectional Survey

**DOI:** 10.2196/17154

**Published:** 2020-09-09

**Authors:** Lisa Tisdale Wigfall

**Affiliations:** 1 Division of Health Education Department of Health and Kinesiology Texas A&M University College Station, TX United States

**Keywords:** eHealth, communication, HIV, disparities, inequalities

## Abstract

**Background:**

Providing HIV/STD testing and prevention education, medical and nonmedical case management, housing assistance, transportation services, and patient navigation are just a few examples of how community-based HIV/AIDS service organizations will help the United States realize the goals of the updated National HIV/AIDS Strategy.

**Objective:**

In this study, the aim was to assess electronic data security confidence level, electronic communication behaviors, and interest in using eHealth communication tools with clients of staff at community-based HIV/AIDS service organizations.

**Methods:**

Staff were recruited from 7 community-based HIV/AIDS service organizations in the southern United States (3 in South Carolina and 4 in Texas). The principal investigator used state department of health websites to identify community-based HIV/AIDS service organizations. Staff were included if they provided HIV/STD prevention education to clients. A recruitment letter was sent to community-based HIV/AIDS service organization leaders who then used snowball sampling to recruit eligible staff. Chi-square tests were used.

**Results:**

Among staff (n=59) who participated in the study, 66% (39/59) were very or completely confident that safeguards are in place to keep electronically shared information from being seen by other people; 68% (40/59) used email, 58% (34/59) used text messages, 25% (15/59) used social media, 15% (9/59) used a mobile app, 8% (5/59) used web-enabled videoconferencing, and 3% (2/59) used other tools (eg, electronic medical record, healthnavigator.com website) to communicate electronically with their clients. More than half were very interested in using eHealth communication tools in the future for sharing appointment reminders (67%, 38/59) and general health tips (61%, 34/59) with their clients. Half were very interested in using eHealth communication tools in the future to share HIV medication reminders with their clients (50%, 29/59). Forty percent (23/59) were very interested in using eHealth communication tools to share vaccination reminders with their clients.

**Conclusions:**

Community-based HIV/AIDS service organization staff had some level of confidence that safeguards were in place to keep electronically shared information from being seen by other people. This is critically important given the sensitivity of the information shared between community-based HIV/AIDS service organization staff and their clients, and because many staff were very interested in using eHealth communication tools with their clients in the future. It is very likely that eHealth communication tools can be used in community settings to improve health outcomes across the HIV care continuum; in the interim, more research is needed to better understand factors that may facilitate or impede community-based HIV/AIDS service organization staff use and client acceptability.

## Introduction

### Background

The 4 main goals of the updated National HIV/AIDS Strategy [1] are reducing new HIV infections, increasing access to care and improving health outcomes for people living with HIV, reducing HIV-related disparities and health inequities, and achieving a more coordinated national response to the HIV epidemic. Providing HIV/STD testing and prevention education, medical and nonmedical case management, housing assistance, transportation services, and patient navigation are just a few examples of how community-based HIV/AIDS service organizations will help the United States realize its vision to

become a place where new HIV infections are rare, and when they do occur, every person, regardless of age, gender, race/ethnicity, sexual orientation, gender identity, or socio-economic circumstance, will have unfettered access to high quality, life-extending care, free from stigma and discrimination

1

Electronic health (eHealth) is a field in which health service delivery and information sharing are enhanced through the use of the internet and related technologies [2]. A recent study provides the following 3 clearly defined domains of eHealth: (1) health in our hands (using eHealth technologies to monitor, track, and inform health), (2) interacting for health (using digital technologies to enable health communication among practitioners and between health professionals and clients or patients), and (3) data enabling health (collecting, managing, and using health data) [3].

In the field of HIV/AIDS prevention, eHealth communication tools improve health outcomes across the HIV care continuum, which include diagnosis of HIV infection; linkage, engagement, retention in HIV care; and achieving viral suppression [4,5]. Recent studies [6-14] have shown how social media and technologies such as avatar-led and smartphone games can be leveraged to prevent the spread of HIV infection and sexually transmitted diseases, as well as improve HIV-related health outcomes such as diagnosis of HIV infection; linkage, engagement, retention in HIV care; and medication adherence.

Unfortunately, some community-based HIV/AIDS service organization clients will encounter barriers using eHealth communication tools to access health services such as HIV/STD testing and HIV care. While some researchers have studied eHealth literacy, acceptability, adoption, and attitudes among individuals such as people living with HIV and men who have sex with men, the target audience is usually the client and not staff [15-19], though some have studied eHealth adoption and attitudes among health care providers in clinical settings [15].

### Objective

While eHealth communication tools have been studied from the perspective of community-based HIV/AIDS service organization clients, little is known about electronic communication beliefs, behaviors, and attitudes of community-based HIV/AIDS service organization staff. This is unfortunate because researchers have shown that community-based HIV/AIDS service organization staff are a trusted source of information about health or medical topics, in addition to their primary role aimed at improving health outcomes along the HIV care continuum [20]. In this study, the principal investigator assessed staff confidence in electronic communication data security, electronic communication behaviors with clients, and attitudes toward using eHealth communication tools with their clients in the future among community-based HIV/AIDS service organization staff. The long-term goal is to build the capacity of community-based organizations to leverage their ability to reach marginalized vulnerable populations with eHealth communication tools that can improve their overall health and well-being, especially among people living with HIV who are underrepresented (compared to men who have sex with men) such as women, transgender persons, and older adults.

## Methods

### Data Collection

The principal investigator (LTW) used state department of health websites to identify community-based HIV/AIDS service organizations. A recruitment email was sent to community-based HIV/AIDS service organization leaders who were asked to invite their eligible staff to participate in the survey. Staff were included if they provided HIV/STD prevention education to clients. No other inclusion or exclusion criteria were used. Positions that were commonly held by participants were case manager, counselor, patient navigator (including linkage specialist or care coordinator), social worker, program coordinator or manager, prevention coordinator, and outreach specialist.

Staff were recruited from 7 community-based HIV/AIDS service organizations in nonclinical urban settings located in the southern United States. In May 2016 and June 2016, the principal investigator (LTW) conducted a paper and pencil survey with from staff at 3 community-based HIV/AIDS service organizations located in South Carolina. Approximately two-and-a-half to three years later (October 2018 to December 2018 and May 2019) when the principal investigator relocated to Texas, the survey was converted to an online format (Qualtrics XM) and distributed to staff at 4 community-based HIV/AIDS service organizations located in Texas. These study details have been previously published [20-22].

### Measures

#### Sociodemographic Characteristics

To describe the sociodemographic characteristics of community-based HIV/AIDS service organization staff, age, sex, sexual orientation, marital status, race/ethnicity, and education were assessed. See Multimedia Appendix 1 for more details.

#### Electronic Data Security Confidence Level

To assess electronic data security confidence level, community-based HIV/AIDS service organization staff were asked, “How confident are you that safeguards are in place to keep electronically shared information from being seen by other people?” This question was adapted from the following Health Information National Trends Survey validated question [23]: “How confident are you that safeguards (including the use of technology) are in place to protect your medical record from being seen by people who aren’t permitted to see them?” Participants responded using a 5-point Likert scale. Response options were coded as 0 (not at all confident) to 4 (completely confident). For analysis, this item was recoded into a dichotomous variable where 1 (completely or very confident) and 0 (somewhat or a little confident). See Multimedia Appendix 2 for more details.

#### Electronic Communication With Clients

A dichotomous (yes or no) variable was used to assess the following modes that community-based HIV/AIDS service organization staff used to communicate electronically with their clients: email, text message, social media, web-enabled videoconference, mobile app, or something else. See Multimedia Appendix 2 for more details. A composite score was calculated so that a higher value represented the modes of electronic communication that staff used more.

#### Future eHealth Communication Tools With Clients

To assess interest in using eHealth communication tools with clients in the future, community-based HIV/AIDS service organization staff were asked, “How interested are you in electronically sharing the following types of health-related information with your clients: appointment reminders, HIV medication reminders, vaccination reminders, and general health tips?” Response options ranged from 1 (not at all interested) to 4 (very interested). See Multimedia Appendix 2 for more details. For analysis, this item was recoded into a dichotomous variable, 1 (very interested) or 0 (somewhat, a little, or not all interested). A composite score was calculated so that a higher value represented more modes of eHealth communication that staff were interested in using to communicate with their clients in the future.

### Data Analysis

Data for this cross-sectional study were analyzed using Stata/SE (version 16.1; StataCorp LLC). Median (range) or frequency (percentage) were calculated for sociodemographic characteristics, electronic data security confidence level, electronic communication with clients, and future eHealth communication with clients. Chi-square tests were performed to examine bivariate associations between electronic data security confidence level and the following: sociodemographic characteristics, electronic communication with clients, and future eHealth communication with clients. *P*<.05 was used to determine statistical significance.

### Ethics

This study was reviewed and approved by the institutional review boards at the University of South Carolina and Texas A&M University.

## Results

### Participants

Table 1 shows the sociodemographic characteristics and median (range) values of the key measures for staff (n=59) at community-based HIV/AIDS service organizations located in the southern United States. The median age for staff was 44 years old (range: 21-70, n=56). Most were younger than 50 years old (36/59, 64%). More than half were female (35/59, 59%), and 41% (24/59) were a sexual minority. Only 10% (6/59) were Hispanic; the majority were non-Hispanic Black or African American (44/59, 74%). All had at least a high school diploma or GED, with 46% (27/59) having earned an undergraduate degree and 25% (15/59) having earned a master’s degree.

**Table 1:** Sociodemographic and eHealth communication characteristics of staff at community-based HIV/AIDS service organizations located in the southern United States (n=59).

Characteristic		Value	
Age in years (n=56), median (range)		44 (21-70)	
**Sex (n=59), n (%)**			
	Male		24 (41)
	Female		35 (59)
**Sexual orientation (n=59), n (%)**			
	Straight, that is, not lesbian or gay		35 (59)
	Lesbian or gay		20 (34)
	Bisexual		3 (5)
	Preferred not to say		1 (2)
**Marital status (n=59), n(%)**			
	Single, never been married		29 (49)
	Married or living as married		24 (41)
	Divorced		4 (7)
	Separated		2 (3)
**Race/ethnicity (n=59), n (%)**			
	Hispanic		6 (10)
	Black or African American		44 (74)
	White		7 (12)
	Asian		1 (2)
	Multiple		1 (2)
**Education (n=59), n (%)**			
	High school diploma/GED/some college		17 (29)
	Undergraduate degree		27 (46)
	Postgraduate degree		15 (25)
Electronic data security confidence level (n=59), median (range)		3 (1-4)	
Electronic communication with clients^a^ (n=59), median (range)		2 (0-5)	
Future eHealth communication with clients^b^ (n=56), median (range)		2 (0-4)	

^a^Email, text, social media, web-enabled videoconference, mobile app, something else (eg, electronic medical record, healthnavigator website).

^b^Appointment, medication, vaccination reminders, or general health tips.

### Electronic Data Security Confidence Level

All were at least a little confident that safeguards are in place to keep electronically shared information from being seen by other people—Table 1 shows that the median confidence level was 3 (range 1-4) (ie, 3 indicates very confident). Among 59 staff, 66% (39/59) were very or completely confident and 34% (20/59) were somewhat or a little confident that safeguards are in place to keep electronically shared information from being seen by other people. Table 2 shows that there were no statistically significant differences in electronic data security confidence level between community-based HIV/AIDS service organization staff based on sociodemographic characteristics (age: *P*=.73, sex: *P*=.23, sexual orientation: *P*=.63, marital status: *P*=.53, race/ethnicity: *P*=.77, education: *P*=.82).

**Table 2:** Bivariate associations between sociodemographic characteristics and eHealth communication modes of staff at community-based HIV/AIDS service organizations by electronic data security confidence level (n=59).

Characteristic				All (n=59), n (%)	Electronic data security confidence level			*P* value
					Completely or very confident, (n=39), n (%)	Somewhat or a little confident (n=20), n (%)		
**Sociodemographic**								
	Age in years^a^, <50		36 (64)		25 (66)	11 (61)	.73	
	Sex, female		35 (59)		21 (54)	14 (70)	.23	
	Sexual orientation, sexual minority^b^		24 (41)		15 (38)	9 (45)	.63	
	Marital status, unmarried^c^		35 (59)		22 (56)	13 (65)	.53	
	**Race/ethnicity**						.77	
		Hispanic	6 (10)		3 (8)	3 (15)		
		Black or African American	44 (74)		29 (74)	15 (75)		
		White	7 (12)		5 (12)	2 (10)		
		Asian	1 (2)		1 (3)	0		
		Multiple	1 (2)		1 (3)	0		
	**Education**						.82	
		High school/GED/some college	17 (29)		12 (31)	5 (25)		
		Undergraduate degree	27 (46)		18 (46)	9 (45)		
		Postgraduate degree	15 (25)		9 (23)	6 (30)		
**eHealth Communication**								
	Electronic communication with clients, any mode^d^		52 (88)		33 (85)	19 (95)	.24	
	**Future eHealth communication with clients, very interested**							
		Appointment reminders^e^	38 (67)		26 (68)	12 (63)	.69	
		Medication reminders^f^	29 (50)		21 (55)	8 (40)	.27	
		Vaccination reminders^e^	23 (40)		18 (47)	5 (26)	.13	
		General health tips^a^	34 (61)		21 (57)	13 (68)	.40	

^a^Data were missing for 3 individuals.

^b^Lesbian or gay, bisexual, or preferred not to say.

^c^Divorced, separated, or single.

^d^Email, text, social media, web-enabled videoconference, mobile app, something else (eg, electronic medical record, healthnavigator website).

^e^Data were missing for 2 individuals.

^f^Data were missing for 1 individual.

### Electronic Communication With Clients

The majority of staff had used at least one form of electronic communication with their clients. Table 1 shows that the median number of electronic communication modes was 2 (range 0-5). More than half of staff had used email (40/59, 68%) and text messages (34/59, 58%) to communicate electronically with their clients. While 15 out of 59 staff at community-based HIV/AIDS service organizations (25%) who participated in our study had used social media (eg, Facebook, Twitter, Instagram, Snap Chat) to communicate electronically with their clients, very few had used a mobile app (eg, What’s App; 9/59, 15%), web-enabled videoconference (eg, FaceTime, Skype; 5/59, 8%), or something else (ie, electronic medical record, healthnavigator website; 2/59, 3%). There were no statistically significant differences in the use of any mode of electronic communication with clients between staff who were completely or very confident that safeguards are in place to keep electronically shared information from being seen by other people when compared to those who were somewhat or a little confident (*P*=.24) (Table 2). Among the few (7/59, 12%) who did not communicate electronically with their clients, phone and mail were the preferred modes of communication.

### Future eHealth Communication With Clients

**Figure 1:**
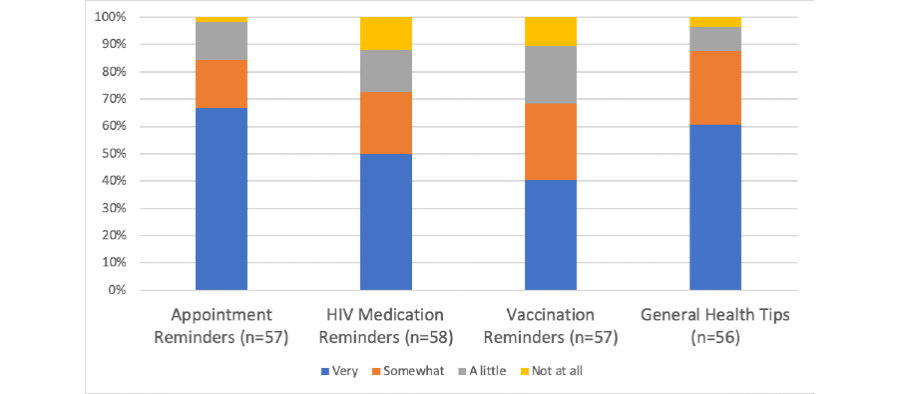
Community-based HIV/AIDS service organizations staff interest in using eHealth communication tools with clients in the future.

Many staff were interested in using eHealth communication tools with their clients in the future. The median number of eHealth communication modes that community-based HIV/AIDS service organization staff were interested in using with their clients in the future was 2 (range 0-4) (Table 1). Figure 1 and Table 2 show that more than half of staff were very interested in using eHealth communication tools in the future for sharing appointment reminders (38/57, 67%) and general health tips (34/56, 61%) with their clients. Almost half were very interested in using eHealth communication tools in the future to share HIV medication reminders with their clients (29/58, 50%), and 40% (23/57) were interested in using eHealth communication tools in the future to share vaccination reminders with their clients. There were also no statistically significant differences in interest in using eHealth communication tools with clients in the future between staff who were completely or very confident that safeguards are in place to keep electronically shared information from being seen by other people compared to those who were somewhat or a little confident (appointment reminders: *P*=.69, HIV medication reminders: *P*=.27, vaccination reminders: *P*=.13, general health tip: *P*=.40) (Table 2).

## Discussion

### Principal Findings

The sample of community-based HIV/AIDS service organization staff had some level of confidence that safeguards were in place to keep electronically shared information from being seen by other people. In fact, the majority were completely or very confident about the security of electronically shared information. Having at least some level of confidence about data security could probably be attributed to the fact that many of the community-based HIV/AIDS service organization staff in the sample had used multiple modes of electronic communication with their clients. Only a few preferred to use phone or mail to communicate with their clients. Additionally, many community-based HIV/AIDS service organization staff were very interested in using eHealth communication tools to remind their clients about appointments, medications, and vaccinations, as well as to share general health tips with them.

According to recent data from the Pew Research Center, 90% of adults are online, which includes 81% who are online daily [24]. Of the adults who are online daily, 45% go online several times a day, and 28% are almost constantly online [24]. Although most adults are online, disparities exist among offline adults despite efforts to close the digital divide. Offline adults are more likely to be Hispanic or Black, older (ie, aged ≥65 years), lower socioeconomic status (ie, annual household income less than US $30,000; less than a high school diploma), and from rural communities [25]. People living with HIV share similar sociodemographic characteristics of offline adults such as racial or ethnic minority and lower socioeconomic status [26]. Unfortunately, these are the same characteristics of individuals who have been experiencing the digital divide in HIV/AIDS care for decades [27]. Chiasson and colleagues [28] concluded from their research study that

HIV prevention and care programs using digital media have great potential to cost-effectively meet the complex needs of diverse and often underserved populations living with or at high risk of HIV

28

In this study, among the staff recruited from community-based HIV/AIDS service organizations located in South Carolina and Texas, more than half used email and text message to communicate with their clients. In addition to the vast number of US adults who are online, another contributing factor to this study's findings is the fact that most people living with HIV are men who have sex with men who, studies have shown, go online for health information and social networking [10]. Additionally, the fact that most of the community-based HIV/AIDS service organization staff who participated in the study were completely or very confident that electronically shared information was protected and private is also a contributing factor to electronic communication with clients, and interest in eHealth communication with clients in the future.

### Study Strengths and Limitations

This study adds to the scientific literature about eHealth communication among staff at community-based HIV/AIDS service organizations; however, there were some limitations. This cross-sectional study used self-reported data to describe eHealth communication attitudes, beliefs, and behaviors. The findings are not generalizable to community-based HIV/AIDS service organizations either across the southern United States or the entire United States since this study only included staff at organizations that were located in only 2 states in the southern US Census region. Additionally, the author was unable to determine the response rate because the total number of employees working at each organization was not obtained from all of the community-based HIV/AIDS service organization leaders.

### Conclusion

Staff at community-based HIV/AIDS service organizations will play a critical role in moving us forward toward realizing the goals of the National HIV/AIDS Strategy, which are aimed at improving health outcomes across the HIV care continuum. Many of the staff at community-based HIV/AIDS service organizations who participated in this study were either already using electronic communication or were very interested in using eHealth communication tools with their clients in the future. In fact, only 12% of staff had not used any form of electronic communication. More research is needed to better understand how to build the capacity of community-based HIV/AIDS service organizations and their staff to help their clients overcome communication inequalities that have the potential to negatively affect HIV/AIDS-related outcomes in these vulnerable populations.

### Public Health Implications

The current pandemic is a prime example of how eHealth communication tools can be used to improve health outcomes along the HIV care continuum. Although many organizations were forced to close during shelter-in-place orders that were issued in response to rising coronavirus disease 2019 (COVID-19) rates, as with all individuals, the health needs of people living with HIV continued. For individuals for whom community-based HIV/AIDS service organization staff are the only trusted source of information about health or medical topics, electronic communication has become a necessity.

## Appendix

Multimedia Appendix 1 Sociodemographic characteristics questionnaire.

Multimedia Appendix 2 eHealth communication questionnaire.

## Abbreviations

AIDS: acquired immunodeficiency syndrome
HIV: human immunodeficiency virus
STD: sexually transmitted disease
