# Supplementary material for: eHealth Communication With Clients at Community-Based HIV/AIDS Service Organizations in the Southern United States: Cross-Sectional Survey
Source: JMIR Form Res. 2020 Sep 9;4(9):e17154. doi: 10.2196/17154 (PMC7511854; doi:10.2196/17154)
Supplement: Multimedia Appendix 1 [file formative_v4i9e17154_app1.pdf]

Appendix 1. Sociodemographic Characteristics Questionnaire

Sociodemographic Characteristics

What is your date of birth? (MM/DD/YYYY)

____

Which of the following best describes your sex at birth?

- ○ Female (1)
- ○ Male (0)

Which of the following best describes your sex now?

- ○ Female (1)
- ○ Male (0)

Which of the following best represents how you think of yourself?

- ○ Lesbian or gay (1)
- ○ Straight, that is, not lesbian or gay (2)
- ○ Bisexual (3)
- ○ Something else (4)
- ○ I don't know (5)

What is your marital status?

- Married (1)
- Living as married (2)
- Divorced (3)
- Widowed (4)
- Separated (5)
- Single, never been married (6)

Are you Hispanic or Latina/o?

- Yes (1)
- No (0)

What is your race?
Mark all that apply.

- Black or African American (1)
- White (2)
- Other (please specify how you would describe your race below): (3) ____

What is the highest grade or level of schooling you completed?

○ I did not have a high school diploma or GED (please specify highest grade completed below): (1) ____

○ High school diploma (2)

○ GED (3)

○ Post-high school training other than college (e.g., vocational or technical school) (4)

○ Less than one year of college (5)

○ More than one year of college (6)

○ College graduate (undergraduate degree) (7)

○ Other (please specify the highest level of schooling you completed below): (8)
____
