# Supplementary material for: eHealth Communication With Clients at Community-Based HIV/AIDS Service Organizations in the Southern United States: Cross-Sectional Survey
Source: JMIR Form Res. 2020 Sep 9;4(9):e17154. doi: 10.2196/17154 (PMC7511854; doi:10.2196/17154)
Supplement: Multimedia Appendix 2 [file formative_v4i9e17154_app2.pdf]

Appendix 2. eHealth Communication Questionnaire

Using the Internet to Find and Share Health Information

How confident are you that safeguards are in place to keep electronically shared information from being seen by other people?

- ○ Completely confident (1)
- ○ Very confident (2)
- ○ Somewhat confident (3)
- ○ A little confident (4)
- ○ Not at all confident (5)

Note: The above question was adapted from the following NCI Health Information National Trends Survey question (see **D2** below) that was asked on the HINTS 4, Cycle 1 (2011), HINTS 4, Cycle 4 (2014), HINTS 4, Cycle 1 (2017), and HINTS 4, Cycle 2 (2018): *How confident are you that safeguards (including the use of technology) are in place to protect your medical record from being seen by people who aren't permitted to see them?* The five-point Likert scale from the health/cancer information seeking self-efficacy question (see **A6** below) was used instead of the following 3-point Likert scale: 1-very confident, 2-somewhat confident, and 3-not confident.

A6. Overall, how confident are you that you could get advice or information about cancer if you needed it? CancerConfidentGetHealthInf

- 1 Completely confident
- 2 Very confident
- 3 Somewhat confident
- 4 A little confident
- 5 Not confident at all

D2. How confident are you that safeguards (including the use of technology) are in place to protect your medical records from being seen by people who aren't permitted to see them? ConfidentInfoSafe

- 1 Very confident
- 2 Somewhat confident
- 3 Not confident

Source: NCI, HINTS 5, Cycle 2 (2018). Available at https://hints.cancer.gov/.

Have you ever communicated electronically with your clients or members using...
Mark all that apply.

	Yes (1)	No (2)
Email? (1)	☐	☐
Text message? (2)	☐	☐
Social media (e.g., Facebook, Twitter, Instagram, Snap Chat)? (3)	☐	☐
Web-enabled video conference (e.g., FaceTime, Skype)? (4)	☐	☐
A mobile app (e.g., What's App)? (5)	☐	☐
Something else (please specify below): (6)	☐	☐

How interested are you in electronically sharing the following types of health-related information with your clients or members...

	Not at all (1)	A little (2)	Somewhat (3)	Very (4)
Appointment reminders? (1)	○	○	○	○
HIV medication reminders? (2)	○	○	○	○
Vaccination reminders? (3)	○	○	○	○
General health tips? (4)	○	○	○	○
